# Supplementary material for: Optimal distribution-preserving downsampling of large biomedical data sets (opdisDownsampling)
Source: PLoS One. 2021 Aug 5;16(8):e0255838. doi: 10.1371/journal.pone.0255838 (PMC8341664; doi:10.1371/journal.pone.0255838)
Supplement: S1 File — The supplementary information comprises (i) probabilistics of the possible combinations in class-proportional downsampling scenarios and (ii) the detailed description of the PCA based reconstruction of data including relevant equations. (PDF) [file pone.0255838.s001.pdf]

Supplementary information

Possible combinations of instances in class proportional sampling

The number of possible combinations of *r* instances of a data set with *n* cases equals the number of

$$Combinations\ = \binom{n}{r} = \frac{n!}{r! \cdot (n-r)!}\ .$$

If a fraction q in [0,1] of a data set is drawn and the data set contains $m > 1$ classes ($k_1,\ldots,k_m$) with weights w_k adding up to a value of 1, the number of instances drawn from each class equals to $r_k = w_k \cdot n \cdot q$, and the number of possible combinations per class is given by

$$Combinations_{\ k} = \frac{(n \cdot w_k)!}{(w_k \cdot n \cdot q)! \cdot \big(w_k \cdot n \cdot (1-q)\big)!}$$

and the absolute number of possible combinations for subsamples of size q preserving the class proportion is calculated to the product $Combinations = \prod_{k=1}^{m} Combinations_{\ k}$.

The probability of each a particular combination *C* among the instances in a class *k* is the reciprocal of that value, i.e.,

$$P(C)\ = \frac{(w_k \cdot n \cdot q)! \cdot \big(w_k \cdot n \cdot (1-q)\big)!}{(n \cdot w_k)!}.$$

In uniformly distributed sampling from a data set with n values x in the range [a,b], the probability of drawing a particular value is given by

$$P(x)\ = \begin{cases} \frac{1}{n}\ for\ a \le x \le b \\ 0\ for\ x < a\ |\ x > b \end{cases}.$$

In class-proportional sampling, the probability of sampling a particular combination of cases follows from the probability of the combinations within each class $k_1,\ldots,k_m$, and the equal probability of the presence of each class in the final sample, i.e.,

$$P(k) = \prod_{k=1}^{m} \frac{(w_k \cdot n \cdot q)! \cdot (w_k \cdot n \cdot (1-q))!}{(n \cdot w_k)!}.$$

Depending on the fraction class-proportionally sampled from the data set, this possibly leads to a huge number of different combinations of instances that can be drawn.

PCA based reconstruction of data

Dimensionality reduction was achieved by performing a principal component analysis (PCA) of the downsampled data X_{sample}. Prior to transformation, the data was centered on the coordinate origin:

$$\boldsymbol{X} = \boldsymbol{X}_{sample} - \boldsymbol{\mu}$$

Where $\boldsymbol{X}_{sample}$ is a $(qn) \cdot p$ matrix with qn rows (number of instances) and p columns (number of features). Centering is achieved by row wise subtracting the vector of the feature averages $\boldsymbol{\mu}$ with p entries. The actual transformation is performed by

$$\boldsymbol{X}_{proj} = \boldsymbol{XV}$$

Where $\boldsymbol{X}_{proj}$ is the projected $(qn) \cdot p$ matrix and $\boldsymbol{V}$ is a $p \cdot p$ matrix that comprises the unit vectors that define the principal components (PCs) determined via singular value decomposition. Dimensionality reduction is performed selecting the x columns of $\boldsymbol{V}$ which passed the Kaiser-Guttman criterion. This results in the $p \cdot x$ matrix $\boldsymbol{V}_{reduced}$ (with $x \leq p$). The PCA results were then used to predict the remaining data in the original dataset $\boldsymbol{X}_{remaining}$, a $(1-q)n \cdot p$ matrix.

$$\boldsymbol{X}_{recosample} = (\boldsymbol{X}_{remaining}\boldsymbol{V})\boldsymbol{V}_{reduced}^{T} + \boldsymbol{\mu}$$

Thus, the reconstruction MSE between $\boldsymbol{X}_{remaining}$ and $\boldsymbol{X}_{recosample}$ calculates to:

$$MSE = \frac{\sum_{i=1}^{(1-q)n} \sum_{j=1}^{p} \left(\boldsymbol{X}_{recosample}[i,j] - \boldsymbol{X}_{remaining}[i,j] \right)^2}{(1-q)n \cdot p}$$
